# Sex and Age Differences in Motion Sickness in Rats: The Correlation with Blood Hormone Responses and Neuronal Activation in the Vestibular and Autonomic Nuclei

**DOI:** 10.3389/fnagi.2017.00029

**Published:** 2017-02-15

**Authors:** Wei Zhou, Junqin Wang, Leilei Pan, Ruirui Qi, Peng Liu, Jiluo Liu, Yiling Cai

**Affiliations:** Department of Nautical Injury Prevention, Faculty of Navy Medicine, Second Military Medical University, ShanghaiChina

**Keywords:** motion sickness, sex and age differences, behavior response, stress hormone, Fos protein

## Abstract

Many studies have demonstrated sex and age differences in motion sickness, but the underlying physiological basis is still in controversy. In the present study, we tried to investigate the potential correlates of endocrine and/or neuronal activity with sex and age differences in rats with motion sickness. LiCl-induced nausea symptom was evaluated by conditioned gaping. Motion sickness was assessed by measurement of autonomic responses (i.e., conditioned gaping and defecation responses), motor impairments (i.e., hypoactivity and balance disturbance) after Ferris wheel-like rotation, and blood hormone levels and central Fos protein expression was also observed. We found that rotation-induced conditioned gaping, defecation responses and motor disorders were significantly attenuated in middle-aged animals (13- and 14-month-age) compared with adolescents (1- and 2-month-age) and young-adults (4- and/or 5-month-age). LiCl-induced conditioned gapings were also decreased with age, but was less pronounced than rotation-induced ones. Females showed greater responses in defecation and spontaneous locomotor activity during adolescents and/or young-adult period. Blood adrenocorticotropic hormone and corticosterone significantly increased in 4-month-old males after rotation compared with static controls. No significant effect of rotation was observed in norepinephrine, epinephrine, β-endorphin and arginine-vasopressin levels. The middle-aged animals (13-month-age) also had higher number of rotation-induced Fos-labeled neurons in the spinal vestibular nucleus, the parabrachial nucleus (PBN), the central and medial nucleus of amygdala (CeA and MeA) compared with adolescents (1-month-age) and young-adults (4-month-age) and in the nucleus of solitary tract (NTS) compared with adolescents (1-month-age). Sex difference in rotation-induced Fos-labeling was observed in the PBN, the NTS, the locus ceruleus and the paraventricular hypothalamus nucleus at 4 and/or 13 months of age. These results suggested that the sex and age differences in motion sickness may not correlate with stress hormone responses and habituation. The age-dependent decline in motion sickness susceptibility might be mainly attributed to the neuronal activity changes in vestibulo-autonomic pathways contributing to homeostasis regulation during motion sickness.

## Introduction

Motion sickness is a common disorder induced by provocative motion of transports and simulators or by weightlessness and virtual reality (Golding and Gresty, 2015). Although several hypotheses have been proposed to explain motion sickness, the precise biological mechanism of the individual difference in motion sickness susceptibility has not been clarified yet (Zhang et al., 2016). Accumulating evidence indicates that sex and age are two main predictors of motion sickness susceptibility in general populations (Golding, 2006). Women always report greater incidence in motion sickness history, (Flanagan et al., 2005) and seem to be more susceptible to seasickness, carsickness, simulator sickness, and visually induced motion sickness than men (Lawther and Griffin, 1988; Turner and Griffin, 1999; Flanagan et al., 2005). However, the hypotheses of motion sickness etiology such as sensory conflict theory cannot explain gender differences in motion sickness. Evidence-based clinical investigations showed that female gender was also a strong risk factor for chemotherapy-induced and postoperative nausea and vomiting (Golding, 2006; Jordan et al., 2014). These observations seem to suggest that there must be anatomical and/or physiological basis underlying high motion sickness susceptibility in females. However, women are more inclined to admit subjective symptoms (such as dizziness, headache, and nausea) and remember past motion sickness experience (Cheung and Hofer, 2002), thus the social and/or psychological factors cannot be ruled out. In addition, motion sickness susceptibility also fluctuates across age. In general, humans around 6 to 7 years of age begin to feel motion sick during traveling (Reason, 1969). The susceptibility peaks around 9 to 10 years and subsequently declines from the teenage years toward adulthood (Turner and Griffin, 1999). However, whether the age-related decline in motion sickness susceptibility can be attributed to habituation induced by repeated motion exposure during aging is still unclear.

Although motion sickness susceptibility-related factors have been widely and frequently studied in humans, the sex and age differences in motion sickness-related behaviors in rodents have not been fully examined yet. In humans, motion sickness is characterized by subjective symptoms such as drowsiness, headache, stomach discomfort, and nausea as well as objective signs such as pallor, thermal effects and vomiting. Retching and vomiting, as the most pronounced symptom of motion sickness, have always been used to assess motion sickness in *Suncus murinus*, cats, dogs, and humans (Conder et al., 2008; Percie du Sert et al., 2010; Lackner, 2014; Lucot, 2016). Although laboratory rodents lack the ability of vomit, the majority of rats are prone to display pica (McCaffrey, 1985; Horn et al., 2013), defecation response (Ossenkopp and Frisken, 1982), as well as spontaneous locomotion reduction during or after motion sickness (McCaffrey, 1985; Ossenkopp et al., 1994). However, pica seems to be a self-medicative behavior rather than a sensitive indicator of motion sickness due to its delay in response to motion stimulation and prolonged recovery after motion sickness habituation in rodents (Li et al., 2008; De Jonghe et al., 2009; Wang et al., 2012). Our previous study showed that scopolamine treatment and bilateral labyrinthectomy completely abolished rotation-induced defecation incontinence and hypoactivity, indicating the reliability of these two motion sickness assays (Wang et al., 2015). Recent studies revealed that the emetic drugs- or motion stimulation-paired context, taste and/or odor can produce a characteristic gaping reaction in rats (Parker and Limebeer, 2006; Limebeer et al., 2008). The finding that labyrinthectomy eliminated body rotation-induced but not lithium-induced conditioned rejection responses supports the validity of a rat model of motion sickness (Cordick et al., 1999; Ossenkopp et al., 2003). In addition to behavioral reactions, motion sickness also induced temporal changes in concentration of blood hormones including adrenocorticotropic hormone (ACTH), corticosterone, norepinephrine, epinephrine, β-endorphin and arginine-vasopressin (AVP) in rodents (Otto et al., 2006; Mo et al., 2012). Furthermore, Fos protein, as an indicator of neuronal activity, might also be a molecular marker for MS development and habituation (Abe et al., 2010; Cai et al., 2010). Studies conducted in a variety of animal species found that provocative motion stimulation enhanced Fos protein expression in vestibulo-autonomic nuclei including the caudal medial and spinal vestibular nucleus (MVN and SpVN), the nucleus of solitary tract (NTS), the parabrachial nucleus (PBN), in the limbic system such as the central and medial nucleus of amygdala (CeA and MeA) and in the areas mediating vestibular stress responses such as the locus ceruleus (LC) and the paraventricular hypothalamus nucleus (PVN) (Nakagawa et al., 2003; Cai et al., 2010; Balaban et al., 2014). Therefore, we hypothesized that the patterns of blood hormone responses and central Fos expression might contribute to sex and age differences in motion sickness.

In this study, we tried to investigate whether there are sex and age differences in motion sickness in rats exposed to Ferris wheel-like rotation. Motion sickness-related gastrointestinal responses were assessed by measuring conditioned gaping and defecation responses; whereas the motor and balance impairments were evaluated using open field test and balance beam test in adolescent, young-adult and middle-aged male and female animals. In order to explore the endocrine and/or neuronal activity correlations of sex and age differences in motion sickness responses, we also examined the blood stress hormone levels and observed Fos protein expression pattern after rotation in male and female rats across ages.

## Materials and Methods

### Animals and Ethics

The Sprague–Dawley rats at approximately 1 and 2 months of age (adolescent), 4 and 5 months of age (young adult), and 13 and 14 months of age (middle-aged adult) were purchased from Shanghai Laboratory Animal Center. The animals were individually housed under a 12 h light:12 h dark cycle (temperature: 22 ± 2°C and lighting: 8:00–20:00) with free access to food and water. All animals were adapted to the lab environment for 2 weeks at room temperature (22–24°C) before initiation of the experiment. All animal protocols and procedures complied with the Guide for the Care and Use of Laboratory Animals (National Research Council, Institute for Laboratory Animal Research, 1996) and were approved by the Ethics Committee for Animal Experimentation of the Second Military Medical University (Shanghai, China). All efforts were made to minimize the number of animals used and their suffering.

### Rotation Device and Procedures

We utilized the whole body Ferris wheel-like rotation which produced resultant centrifugal force composed of linear forces in both vertical and sagittal planes (Crampton and Lucot, 1985). The rotation device and detailed rotation methods were described previously (Cai et al., 2007). Briefly, the animals were placed in plexiglass chambers with the long axis of the body perpendicular to the horizontal rotation rod. The chambers were made of black opaque Plexiglas sides (22.5 cm × 26 cm × 20 cm) with a black lid and a clear Plexiglas bottom. The device started to rotate in a clockwise direction at 16°/s^2^ to reach an angular velocity of 120°/s and then began to decelerate at 48°/s^2^ to reach 0°/s. After a 1 s pause, the container continued to rotate in a counter-clockwise direction in the same manner as above. The clockwise-pause-counterclockwise cycle lasted approximately 21 s. All of the rats in the rotation (Rot) groups received 2 h of rotation stimulation in complete darkness, while the animals in the static control (Sta) groups were kept in the plexiglass chambers near the rotation device when Rot animals were being rotated.

### Experiment Design and Grouping

Three hundred and eighty-four animals were used to test conditioned gaping induced by LiCl-injection or rotation stimulation (32 males and 32 females in each month-age group). Males or females at different month-ages were randomly divided into the LiCl injection group or the saline injection group and the Rot group or the Sta group (*n* = 8 in each group). All animals received three conditioning trials separated by 24 h and one test trial 24 h following the last conditioning treatment.

To observe the defecation response, the spontaneous locomotion activity and the central Fos expression, 107 males and 119 females at different month-ages were used and randomly divided into the Rot group or the Sta group (*n* = 8–12 in each group). Immediately after rotation or static control treatment, the animals were taken out of the plexiglass containers of the rotation device and were tested for spontaneous locomotion activity. The number of fecal granules deposited by each animal in the plexiglass container was also counted. At the end, three rats were randomly selected from the 1-, 4-, and 13-month-age groups and were killed for Fos immunostaining immediately after the spontaneous locomotion test.

To assess the effect of rotation on balance performance across ages, additional 102 males and 113 females were used and randomly divided into the Rot group or the Sta group as above (*n* = 8–10 in each group). All animals received balance beam test immediately after Rot or Sta treatment and blood hormone levels were also measured in animals at 1, 4, and 13 months of age.

### Behavioral Test

#### Conditioned Gaping Test

During each conditioning trial, a 10 ml test tube containing a cotton dental roll saturated with vanilla flavor extract (McCORMICK; 35% alcohol) was attached to a hole at one side of the plexiglass chamber of the rotation device. The Rot animals (paired group) simultaneously exposed to an odor conditioned stimulus and the rotation-induced gastric discomfort as an unconditioned stimulus, while the Sta animals (unpaired group) were only exposed to an odor context without rotation. Animals in lithium chloride (LiCl) group were injected intraperitoneally (4 ml/kg i.p.) with 0.15M LiCl solution (32 mg/kg in each animal) and animals in saline (Sal) group were injected with isotonic saline (20 ml/kg, i.p.) 30 min before being placed into conditioning chamber for 2 h without rotation.

Twenty-four hours following the last conditioning trial, each rat received a 1 h test trial in the distinctive context with the odor present without prior rotation or drug injection, and the orofacial and somatic responses of the rat were recorded from the mirror mounted to the conditioning chamber using a video camera (SONY, HDR-PJ670, Japan). The frequency of the gaping (rapid, large-amplitude opening of the mandible with lower incisors exposed) were recorded by viewing the videotapes at a later time by two raters blind to the experimental grouping and design. The inter-rater reliability was examined for each month-age experiment in both the LiCl and rotation-induced gaping test (inter-rater reliability: *r*_(1,30)_ > 0.95 based on the Cronbach’s α model).

#### Spontaneous Locomotion Activity

Spontaneous locomotion was measured by an animal behavior test system (RD1112-IFO-R-4, Mobiledatum, Shanghai, China). The apparatus consisted of a dark 40 cm × 40 cm × 45 cm rectangular chamber with the floor marked with a 16 × 16 grid. The testing was conducted in a soundproof room. The animal was placed in the center of the chamber and left undisturbed for 5 min. Behavior and locomotion tracking of the animals were recorded by an infrared digital video camera. The body center-point moving duration (s), total distance traveled (cm), mobile frequency and immobile (inactivity) duration (s) during the 5 min observation were measured with commercially available software (EthoVision XT 8.5, Noldus, Netherlands) (Wang et al., 2015). As the Sta control levels fluctuate with age remarkably in both males and females, the change rate of these variables in each Rot animal relative to corresponding Sta control levels in average was calculated.

#### Balance Beam Test

The motor coordination was assessed by measuring the time taken to traverse an elevated (90 cm) narrow wooden beam (2.5 cm × 100 cm) and enter a black plastic box (15 cm × 15 cm × 8 cm) at the opposite end. Three trials were performed immediately after Rot or Sta control treatment and the average time to cross the beam for each animal were used in the statistical analysis. The animals were allowed a 60 s rest between trials to reduce stress and fatigue. Before testing, each animal was trained daily for 5–9 consecutive days in order to achieve a stable performance on the balance beam with the time to cross the beam less than 2.5 s.

### Blood Hormone Measurements

Blood was collected immediately after decapitation and the plasma was separated and stored at -80°C for further analyses. The ACTH, corticosterone, norepinephrine, epinephrine, β-endorphin, and AVP levels were measured by radioimmunoassay following the instructions in the kits generously provided by Prof. Zhao XL at the Second Military Medical University or purchased from North Institute of Biological Technology Co (Beijing, China).

### Immunohistochemistry

Animals were anesthetized with an overdose of sodium pentobarbital (100 mg/kg, i.p.) and perfused transcardially with 100 ml chilled saline, followed by perfusion with 500 ml of 0.1 mol/L phosphate buffer (PB, pH 7.4) containing 4% paraformaldehyde. The brains were removed, postfixed with 4% paraformaldehyde at 4°C for 1 h; they were then placed in 0.1 mol/L PB containing 30% sucrose overnight at 4°C and were cut into 20 μm-thick sections throughout. One out of every three consecutive sections containing the MVN and SpVN (Bregma -11.6 to -12.3 mm), the NTS (Bregma -12.7 to -14.3 mm), the PBN (Bregma -8.7 to -10.0 mm), the LC (Bregma -9.10 to -10.0 mm), the CeA and MeA (Bregma -1.8 to -3.6 mm), and the PVN (Bregma -0.8 to -2.1 mm) was selected for use. The selected sections were washed in 0.01 M phosphate-buffered solution (PBS) (pH 7.4) and incubated in a rabbit anti-Fos IgG (Santa Cruz Biotechnology Inc.; 1:1000) for 24 h at 4°C. After washing in PBS, the sections were incubated in biotinylated goat anti-rabbit IgG (Jackson; 1:200) for 4 h. Fos labeling (Fos-LI) was visualized using ABC immunoperoxidase method according to the manufacturer’s instruction (Vector Laboratories, Burlingame, CA, USA). Some control sections were processed without the primary antibody to rule out non-specific immunostaining in these sections. The number of Fos-LI neurons was counted under a light microscope by a rater who was unaware of the experimental conditions of the rats and the photographs were taken with a digital camera.

### Statistical Analysis

The statistical analysis was conducted with the SPSS v13.0 statistical program and the data are expressed as the mean ± SD. Two-way ANOVA was performed using General Linear Protocol to analyze the main effect of sex, age, LiCl, or Rot and their interactions on behavior responses, blood hormone levels and the number of Fos-LI neurons. Fisher’s LSD *post hoc* analysis was used when a significant main effect or interaction effect was obtained. Statistical significance was judged at *P* < 0.05.

## Results

### Behavioral Changes after Rotation in Males and Females across Ages

**FIGURE 1:**
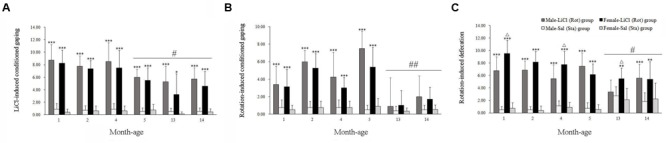
**Conditioned gaping reactions induced by LiCl injection (A)** or rotation **(B)** and defecation responses induced by rotation **(C)** in male and female animals at different ages. Rot, rotation stimulation; Sta, static control treatment. Data are represented as mean ± SEM. ^∗^*P* < 0.05, ^∗∗^*P* < 0.01, ^∗∗∗^*P* < 0.001 compared with corresponding Sta control group; ^Δ^*P* < 0.05 compared with corresponding Male-Rot group; ^#^*P* < 0.05, ^##^*P* < 0.01 compared with other month-age groups.

Two-way ANOVA analysis revealed significant main effects of LiCl [*F*(1,168) = 599.325, *P* < 0.001] and age [*F*(5,168) = 9.727, *P* < 0.001], and an age × LiCl interaction [*F*(5,168) = 6.679, *P* < 0.001] on conditioned gaping. *Post hoc* analysis showed that LiCl injection significantly increased the number of gaping in both male and female animals compared with corresponding Sal controls across all ages (*P* < 0.05 or 0.001; **Figure 1A**). The LiCl-induced gaping responses gradually decreased with age (**Figure 1A**), and were significantly attenuated after 5 months of age compared with other month-age groups in both males and females (*P* < 0.05). **Figure 1B** showed that rotation stimulation also increased conditioned gaping in both males and females at 1, 2, 4, and 5 months of age but not at 13 and 14 months of age [Rot effect: *F*(1,168) = 228.586, *P* < 0.001]. Rot-induced gaping was also significantly decreased at 13 or 14 months of age compared with other month-age groups in both males and females [age effect: *F*(5,168) = 24.834, *P* < 0.001; age × Rot interaction: *F*(5,168) = 18.090, *P* < 0.001; LSD *post hoc*: *P* < 0.01]. Neither LiCl nor Rot treatment induced a sex difference in conditioned gaping across ages [LiCl: sex effect: *F*(1,168) = 2.51, *P* > 0.05; sex × age × LiCl interaction: *F*(5,168) = 0.239, *P* = 0.945; Rot: sex effect: *F*(1,168) = 2.34, *P* > 0.05; sex × age × Rot interaction: *F*(5,168) = 1.096, *P* > 0.05).

There were significant main effects of Rot [*F*(1,202) = 557.542, *P* < 0.001], sex [*F*(1,202) = 4.337, *P* < 0.05], and interaction effects of sex × Rot [*F*(1,203) = 8.604, *P* < 0.01], age × Rot [*F*(5,202) = 15.151, *P* < 0.001] and sex × age × Rot [*F*(5,202) = 2.271, *P* < 0.05] on defecation responses. Rotation stimulation significantly increased defecation responses in both males and females compared with corresponding Sta controls across all ages (*P* < 0.01 or 0.001). Females had higher defecation responses after rotation than males at 1, 4, and 13 months of age (*P* < 0.05; **Figure 1C**), whereas no significant sex difference was observed after Sta treatment. Rot-induced defecation was also significantly declined in males and females at 13 and 14 months of age compared with other month-age groups (*P* < 0.05, **Figure 1C**).

**FIGURE 2:**
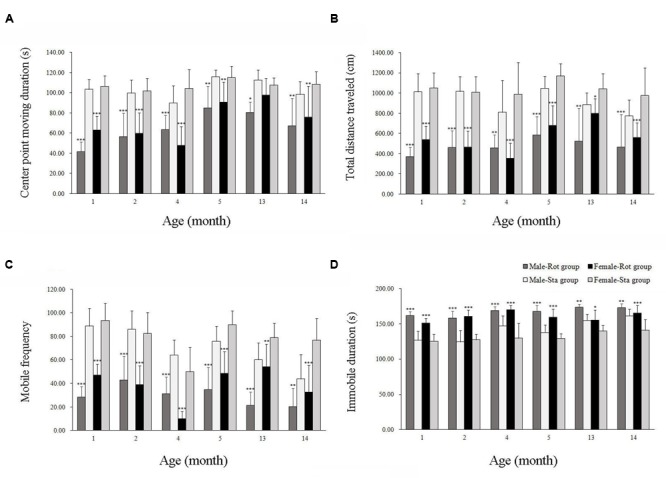
**Spontaneous locomotor activity in an open field after rotation in male and female animals at different ages.** Body center-point moving duration **(A)**, total distance traveled **(B)**, mobile frequency **(C)**, and immobile duration **(D)** were analyzed; Rot, rotation stimulation; Sta, static control treatment. Data are shown as mean ± SEM. ^∗^*P* < 0.05, ^∗∗^*P* < 0.01, ^∗∗∗^*P* < 0.001 compared with corresponding Sta control group.

In the open field test, animals showed a significant difference in center-point moving duration [Rot effect: *F*(1,202) = 101.312, *P* < 0.001; **Figure 2A**], total distance traveled [Rot effect: *F*(1,202) = 17.735, *P* < 0.001; **Figure 2B**], mobile frequency [Rot effect: *F*(1,202) = 320.652, *P* < 0.05; **Figure 2C**], and immobile duration [Rot effect: *F*(1,202) = 309.148, *P* < 0.001; **Figure 2D**] between Rot and Sta controls. Rotation stimulation significantly decreased center point moving duration, total distance traveled and mobile frequency and increased immobile duration in both males and females across all ages compared with corresponding Sta controls.

**Table 1** showed that the change rates of immobile duration were gradually decreased with age in males and females, with the magnitudes significantly lower at 13 and 14 months of age than other month-age groups [age effect: *F*(5,101) = 27.076, *P* < 0.001; *post hoc*: *P* < 0.001]. The change rates of total distance traveled also changed with age with lower responses in males and females at 13 and 14 months of age than those at 1, 2, and 4 months of age [age effect: *F*(5,101) = 9.012, *P* < 0.001; *post hoc*: *P* < 0.05 or 0.001]. Animals at 13 months of age had significantly lower responses to rotation in center-point moving duration [age effect: *F*(5,101) = 7.228, *P* < 0.001; *post hoc*: *P* < 0.001] and mobile frequency [age effect: *F*(5,101) = 3.62, *P* < 0.01; *post hoc*: *P* < 0.05 or 0.01] compared with 1, 2, and 4 months-age groups. Females also had lower change rates than males in center-point moving duration, total distance traveled and mobile frequency than males after rotation at 1 and 13 months of age.

**Table 1:** Change rates of variables in the spontaneous locomotion activity test after rotation in male and female animals at different month-age.

Age (month)	Center-point moving (s)		Total distance traveled (cm)		Mobile frequency		Immobile duration (s)	
	Males	Females	Males	Females	Males	Females	Males	Females
1	60.00 ± 9.24^∗∗∗^	40.86 ± 13.25^∗∗∗^	64.00 ± 9.42^∗∗∗,###^	48.53 ± 12.51^∗∗∗,#^	68.00 ± 10.11^∗∗^	49.60 ± 9.89^∗^	27.39 ± 4.36^∗∗∗,###^	20.60 ± 5.13^∗∗∗,###^
2	43.39 ± 23.59^∗∗∗^	38.44 ± 22.26^∗∗∗^	56.35 ± 16.08^∗∗∗,#^	51.85 ± 17.46^∗∗∗,#^	53.02 ± 12.98^∗^	51.97 ± 21.40^∗∗^	27.11 ± 7.59^∗∗∗,###^	25.52 ± 7.97^∗∗∗,###^
4	49.50 ± 15.89^∗∗∗^	54.23 ± 18.30^∗∗∗^	43.52 ± 15.94^∗,#^	64.39 ± 15.34 ^∗∗∗,###^	51.43 ± 22.09^∗^	70.29 ± 16.60^∗∗^	14.79 ± 3.73^∗∗∗,###^	31.15 ± 4.67^∗∗∗,###^
5	28.62 ± 21.03	21.89 ± 20.73	44.27 ± 19.36	41.23 ± 18.89	48.60 ± 26.82	44.16 ± 22.28	19.93 ± 6.62 ^∗∗∗,###^	21.81 ± 9.93 ^∗∗∗,###^
13	28.86 ± 9.50	9.12 ± 15.65	40.76 ± 10.83	23.26 ± 23.17	44.25 ± 19.11	31.48 ± 24.58	11.91 ± 2.97	10.95 ± 10.62
14	31.47 ± 27.62	30.30 ± 28.60	40.08 ± 23.97	42.97 ± 22.44	53.61 ± 36.01	57.65 ± 29.99	7.18 ± 3.58	17.42 ± 7.64
	**F_sex × age_ (5,101) = 4.068, *P* = 0.002**		**F_sex × age_ (5,101) = 5.700, *P* = 0.0001**		***F*_sex × age_ (5,101) = 6.941, *P* = 0.0001**		***F*_sex × age_ (5,101) = 13.049, *P* = 0.0001**	

*F_sex × age_ represents *F*-values of gender by age interactions in two-way ANOVA; ^∗^*P* < 0.05, ^∗∗^*P* < 0.01, ^∗∗∗^*P* < 0.001 compared with the corresponding 13 months-age group; ^#^*P* < 0.05, ^##^*P* < 0.01 compared with the corresponding 14 months-age group.*

**FIGURE 3:**
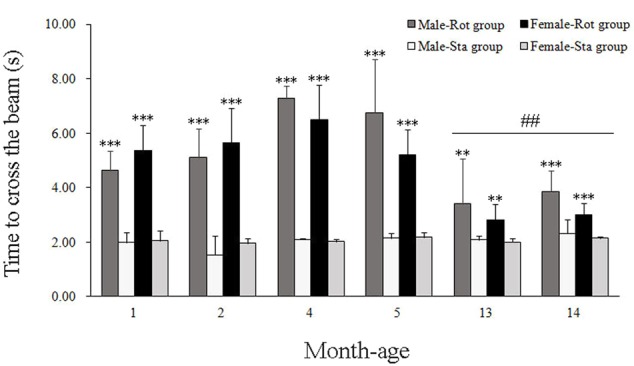
**Motor coordination in balance beam test after rotation in male and female animals at different ages.** Rot, rotation stimulation; Sta, static control treatment. Data are shown as mean ± SEM. ^∗∗^*P* < 0.01, ^∗∗∗^*P* < 0.001 compared with corresponding Sta control group; ^##^*P* < 0.01 compared with other month-age groups.

In balance beam test, rotation stimulation significantly increased the time to cross the beam in male and female animals across all ages compared with Sta controls [Rot effect: *F*(1,191) = 409.336, *P* < 0.001; **Figure 3**]. Males and females at 13 and 14 months of age spent less time to cross the beam after rotation than the animals in other month-age groups [age × Rot interaction: *F*(5,191) = 12.789, *P* < 0.001; *post hoc*: *P* < 0.01 or 0.001], while no significant difference was observed between males and females across ages [sex × Rot: *F*(1,191) = 1.766, *P* > 0.01].

### Blood Hormone Responses after Rotation in Males and Females across Ages

**FIGURE 4:**
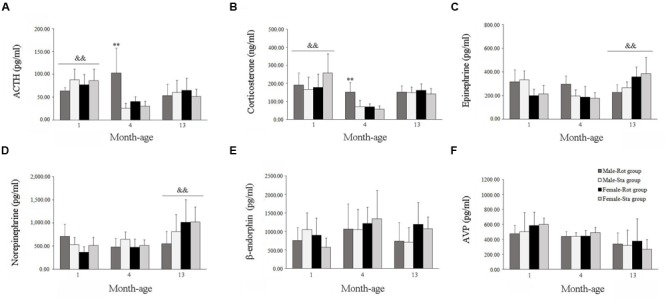
**Blood hormone responses after rotation in male and female animals at different ages.** Rot, rotation stimulation; Sta, static control treatment. Plasma ACTH **(A)**, corticosterone **(B)**, norepinephrine **(C)**, epinephrine **(D)**, β-endophin **(E)**, and AVP **(F)** levels were measured. Data are shown as mean ± SEM. ^∗∗^*P* < 0.01 compared with corresponding Sta control group; ^&&^*P* < 0.01 compared with other month-age groups.

Two-way ANOVA analysis found a significant age effect [*F*(2,108) = 14.067, *P* < 0.001] and significant interaction effects on sex × age [*F*(2,108) = 4.966, *P* < 0.01], age × Rot [*F*(2,108) = 14.188, *P* < 0.001] and sex × age × Rot [*F*(2,108) = 8.164, *P* < 0.01] on plasma ACTH levels. A significant age effect [*F*(2,108) = 14.067, *P* < 0.001] and significant sex × age [*F*(2,108) = 4.966, *P* < 0.01], age × Rot [*F*(2,108) = 14.188, *P* < 0.001] interaction effects on plasma corticosterone levels were also observed. Animals at 1 month of age had higher plasma ACTH and corticosterone levels than those at 4 and 13 months of age (LSD *post hoc*: *P* < 0.01), while rotation stimulation only increased plasma ACTH and corticosterone levels in males at 4 months of age but not in other month-age animals compared with corresponding Sta controls (**Figures 4A,B**). In addition, a significant age effect and a sex × age interaction effect on blood epinephrine [*F*(2,108) = 7.366, *P* < 0.01; *F*(2,108) = 19.100, *P* < 0.001] and norepinephrine [*F*(2,108) = 13.000, *P* < 0.001; *F*(2,108) = 7.651, *P* < 0.01] levels were also revealed. There was a significant age-related increase in blood epinephrine and norepinephrine levels. LSD *post hoc* analysis showed that animals at 13 months of age had higher blood epinephrine and norepinephrine levels than those at 1 and 4 months of age (*P* < 0.01; **Figures 4C,D**). There was no significant Rot effect on blood epinephrine [*F*(1,108) = 0.442, *P* > 0.05], norepinephrine [*F*(1,108) = 0.659, *P* > 0.05] levels in either males or females across ages. No significant main effect of sex, age, or Rot or their interaction effects was observed in blood β-endorphin and AVP levels (**Figures 4E,F**).

### Fos Protein Expression after Rotation in Males and Females across Ages

**FIGURE 5:**
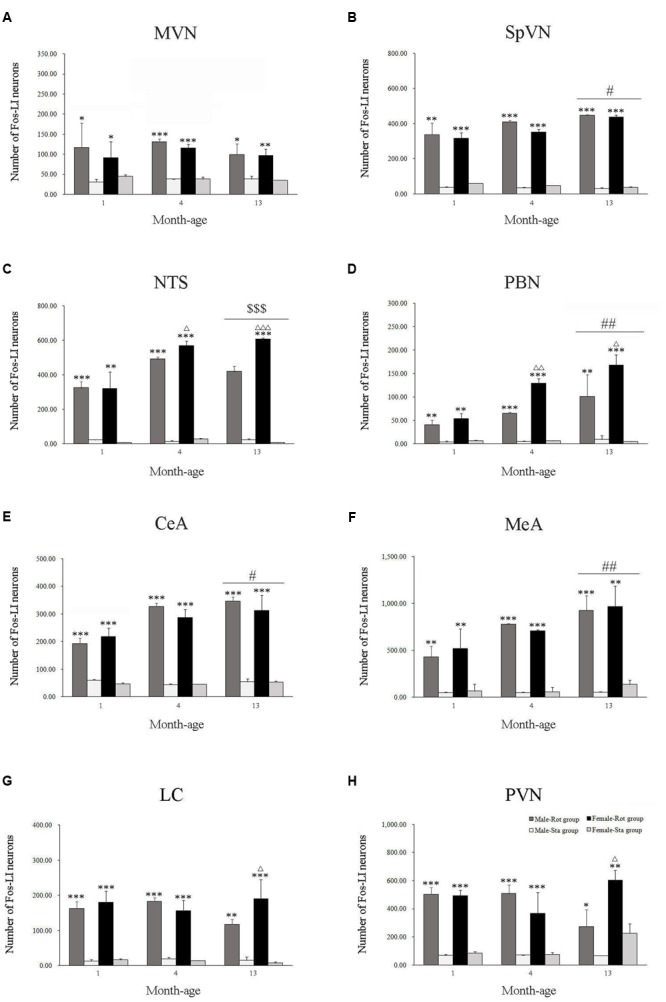
**Number of Fos-LI neurons in the medial vestibular nucleus** (MVN, **A**), the spinal vestibular nucleus (SpVN, **B**), the nucleus of solitary tract (NTS, **C**), the parabrachial nucleus (PBN, **D**), the central amygdala (CeA, **E**), the medial amygdala (MeA, **F**), the locus ceruleus (LC, **G**) and the paraventricular hypothalamus nucleus (PVN, **H**) after rotation in rats at 1, 4, and 13 months-age. Rot, rotation stimulation; Sta, static control treatment. Data are shown as mean ± SEM. ^∗^*P* < 0.05, ^∗∗^*P* < 0.01, ^∗∗∗^*P* < 0.001 compared with corresponding Sta control group; ^Δ^*P* < 0.05, ^ΔΔ^*P* < 0.01, _ΔΔΔ_ P < 0.001 compared with corresponding Male-Rot group; ^#^*P* < 0.05, ^##^*P* < 0.01 compared with other age groups; ^$$$^ *P* < 0.001 compared with 1 month-age groups.

**FIGURE 6:**
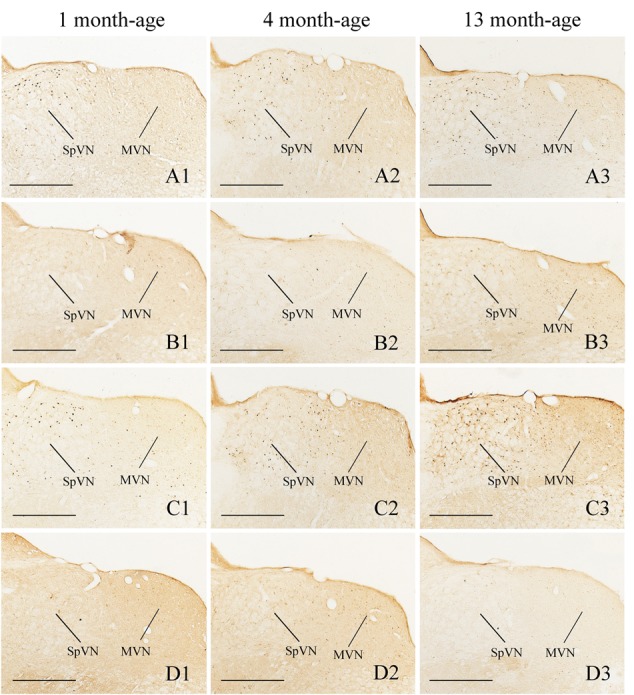
**Representative photomicrographs showing Fos immunolabeling in the MVN and the SpVN in the Male-Rot (A1–A3)**, the Male-Sta **(B1–B3)**, the Female-Rot **(C1–C3)**, and the Female-Sta **(D1–D3)** animals at 1, 4, and 13 months-age. Scale bars = 500 μm.

**FIGURE 7:**
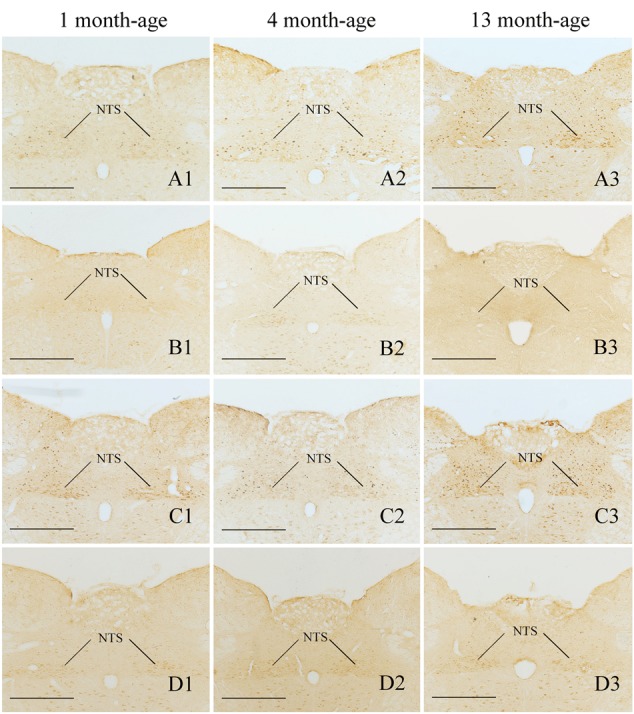
**Representative photomicrographs showing Fos immunolabeling in the NTS in the Male-Rot (A1–A3)**, the Male-Sta **(B1–B3)**, the Female-Rot **(C1–C3)**, and the Female-Sta **(D1–D3)** animals at 1, 4, and 13 months-age. Scale bars = 500 μm.

**FIGURE 8:**
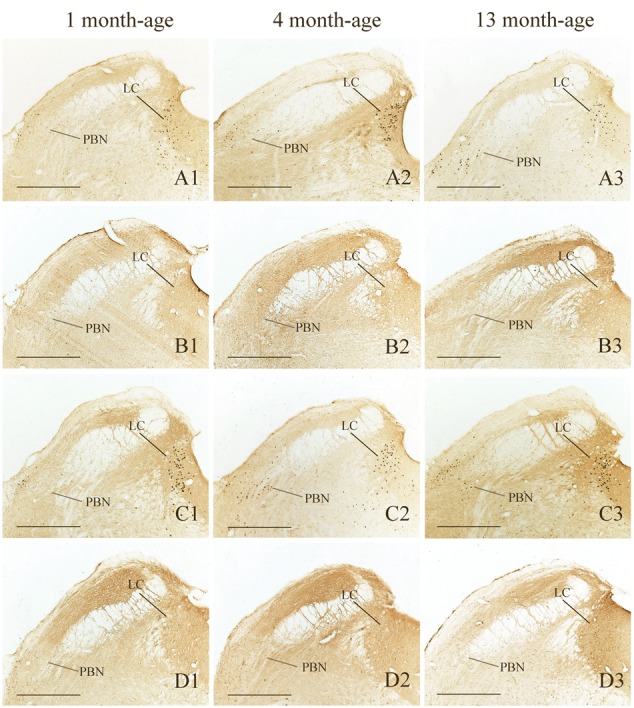
**Representative photomicrographs showing Fos immunolabeling in the PBN and the LC in the Male-Rot (A1–A3)**, the Male-Sta **(B1–B3)**, the Female-Rot **(C1–C3)** and the Female-Sta **(D1–D3)** animals at 1, 4, and 13 months-age. Scale bars = 500 μm.

**FIGURE 9:**
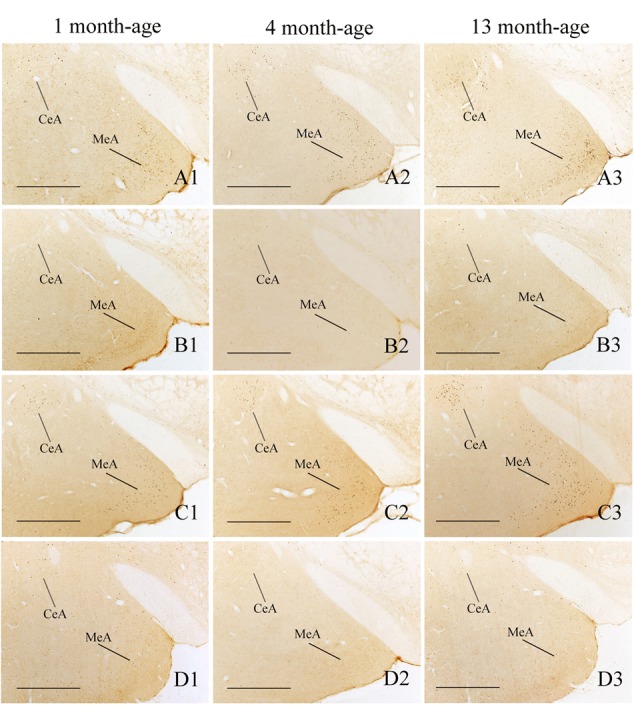
**Representative photomicrographs showing Fos immunolabeling in the central amygdala (CeA) and the medial amygdala (MeA) in the Male-Rot (A1–A3)**, the Male-Sta **(B1–B3)**, the Female-Rot **(C1–C3)**, and the Female-Sta **(D1–D3)** animals at 1, 4, and 13 months-age. Scale bars = 500 μm.

**FIGURE 10:**
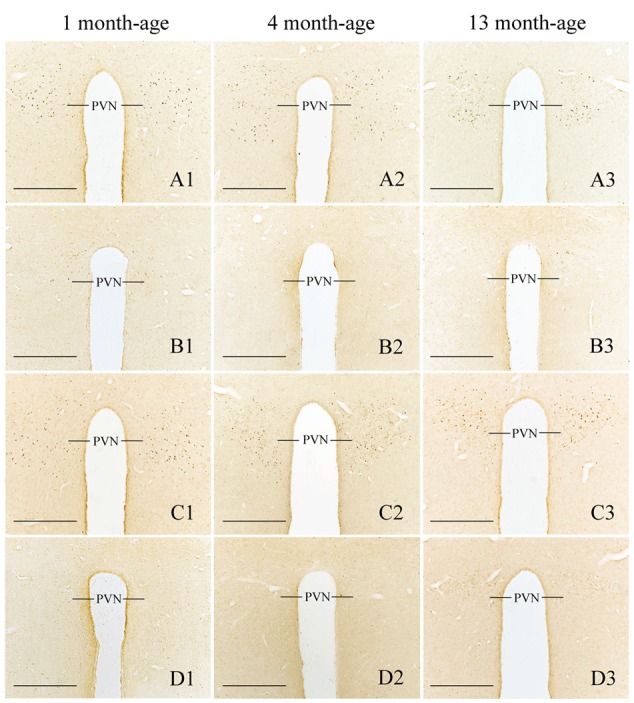
**Representative photomicrographs showing Fos immunolabeling in the PVN in the Male-Rot (A1–A3)**, the Male-Sta **(B1–B3)**, the Female-Rot **(C1–C3)**, and the Female-Sta **(D1–D3)** animals at 1, 4, and 13 months-age. Scale bars = 500 μm.

Compared with Sta controls, rotation stimulation significantly increased the numbers of Fos-LI neurons in the MVN [Rot effect: *F*(1,24) = 173.716, *P* < 0.001], the SpVN [Rot effect: *F*(1,24) = 2077.944, *P* < 0.001], the NTS [Rot effect: *F*(1,24) = 1639.345, *P* < 0.001], the PBN [mainly in external lateral and medial part; Rot effect: *F*(1,24) = 193.819, *P* < 0.001], the LC [Rot effect: *F*(1,24) = 493.006, *P* < 0.001], the CeA [Rot effect: *F*(1,24) = 3131.256, *P* < 0.001], the MeA [Rot effect: *F*(1,24) = 379.485, *P* < 0.001], and the PVN [Rot effect: *F*(1,24) = 503.596, *P* < 0.001] in male and female animals (**Figures 5**–**10**). **Figure 5** shows that the Rot-induced Fos-LI neurons were also increased with age in the SpVN [age × Rot interaction: *F*(2,24) = 24.932, *P* < 0.001], the NTS [age × Rot interaction: *F*(2,24) = 36.993, *P* < 0.001], the PBN [age × Rot interaction: *F*(2,24) = 29.915, *P* < 0.001], the CeA [age × Rot interaction: *F*(2,24) = 10.647, *P* < 0.001] and the MeA [age × Rot interaction: *F*(2,24) = 8.059, *P* < 0.01] in both males and females. Animals at 13 months of age had higher numbers of Fos-LI neurons in the SpVN (LSD *post hoc*: *P* < 0.01; **Figure 6**), the PBN (LSD *post hoc*: *P* < 0.001; **Figure 8**), the CeA (LSD *post hoc*: *P* < 0.05) and the MeA (LSD *post hoc*: *P* < 0.01; **Figure 9**) than other month-age groups and also had higher numbers of Fos-LI neurons in the NTS [sex × Rot interaction: *F*(1,24) = 17.951, *P* < 0.001; sex × age × Rot interaction: *F*(2,24) = 7.005, *P* < 0.01; LSD *post hoc*: *P* < 0.001] than those at 1 month of age (**Figure 6**). The numbers of Fos-LI neurons were also significantly higher in Rot females than Rot males at 4 and 13 months of age in the NTS (LSD *post hoc*: *P* < 0.05 and *P* < 0.001; **Figure 5C**) and the PBN [sex × Rot interaction: *F*(1,24) = 29.915, *P* < 0.001; sex × age × Rot interaction: *F*(2,24) = 7.924, *P* < 0.01; LSD *post hoc*: *P* < 0.01 and *P* < 0.05; **Figure 5D**] and at 13 months of age in the LC [sex × Rot interaction: *F*(1,24) = 6.183, *P* < 0.05; sex × age × Rot interaction: *F*(2,24) = 4.449, *P* < 0.05; LSD *post hoc*: *P* < 0.05; **Figure 5G**] and the PVN [sex × age × Rot interaction: *F*(2,24) = 18.454, *P* < 0.001; LSD *post hoc*: *P* < 0.05; **Figures 5H** and **10**]. No significant difference was observed among Sta males and Sta females across ages within these brain regions.

## Discussion

The present study revealed that both LiCl injection and rotation stimulation significantly induced conditioned gaping (nausea-like behavior) in both male and female animals, but no significant sex differences were found when females were not in menstruation phase. These results are consistent with several laboratory observations in humans which showed no gender difference in susceptibility of circular vection drum or Coriolis cross-coupling stimulation-induced motion sickness indicated by nausea ratings (Cheung and Hofer, 2002; Klosterhalfen et al., 2006). In a study of anurans, parabolic flight significantly induced emesis in the tree frog *R. schlegelii*, but no sex differences was also observed (Naitoh et al., 2001). Nevertheless, studies on motion-induced emesis in adult *Suncus murinus* found more frequent emesis in male animals exposed to 5-min reciprocal shaking, while female animals were more responsive to a much longer stimulation (10 min) with similar amplitude and frequency (Matsuki et al., 1997; Javid and Naylor, 1999). These results clearly demonstrated that females do not always exhibit motion sickness hypersensitivity in either humans or animals. Golding et al. (2005) have reported that motion sickness susceptibility fluctuates through the menstrual cycle in women, and the magnitude of fluctuation in susceptibility across the cycle contributes to around one-third of the overall difference between male and female. Estrous cycle may also influence spontaneous locomotor activity measurements in female rats (Martin and Battig, 1980). The limitation of the present study is that we did not track the estrous cycle and distribution of the estrous cycle was not identified within each group on conditioning and test days. Recently, Cloutier et al. (2017) have confirmed the validity of using conditioned gaping response as an index of nausea in rats. In this study, the conditioning episodes were carried out during the light cycle every 3 days which ensured a different estrous cycle day for females on each of the four conditioning days, and animals in each group were pseudo-randomized across the estrous cycles. They found that female rats had more LiCl-induced gapings and other aversive disgust-related behaviors (forelimb flailing, chin rubbing, and paw treading) than the males. Based on the fact that motion sickness susceptibility might increase during menstruation, it is plausible to presume that female animals within sickness sensitive estrous cycle phase may contribute to high LiCl-induced gaping reactions. Recently, motion sickness-induced hypothermia has been reported in rodents and musk shrews, indicating that thermoregulatory symptoms may serve as physiological correlates of nausea (Nobel et al., 2012; Del Vecchio et al., 2014; Ngampramuan et al., 2014). Gastric myoelectric activity is also associated with nausea in ferrets, *Suncus murinus*, and humans (Kiernan et al., 1997; Percie du Sert et al., 2010; Lu et al., 2014). These physiological variables should be assessed as objective measurements of nausea in the future. Furthermore, previous studies showed that overshadowing by salient stimuli and repetitive context pre-exposure (latent inhibition) can reduce anticipatory nausea in humans and conditioned aversion in rodents, suggesting that there are psychological effects influencing anticipated nausea and vomiting (Klosterhalfen et al., 2005; Hall and Symonds, 2006; Stockhorst et al., 2014). In the meantime, susceptibility to motion sickness was also found to be associated with certain personality characteristics such as trait-anxiety and neuroticism which appeared to be correlated significantly with high motion sickness susceptibility in women (Collins and Lentz, 1977; Bick, 1983; Paillard et al., 2013). Furthermore, manipulation of expectation affected not only motion-induced nausea symptoms but also gastric physiological responses, suggesting an unspecified psychophysiological interaction mechanism responsible for the susceptibility of nausea and emesis to motion stimulation and other nauseogenic stimuli (Williamson et al., 2004; Levine et al., 2006). It is also noteworthy that expectancy for sickness was more effective to enhance tolerance to rotation chair stimulation in men than women (Klosterhalfen et al., 2005; Klosterhalfen et al., 2009). Although women report more overall symptoms of visually induced motion sickness and significantly more gastrointestinal symptoms than males, gastric tachyarrhythmia, as a biological markers of nausea, did not show sex differences accordingly (Jokerst et al., 1999). These evidence suggested that the sex differences in motion sickness may not only arise from menstrual/hormonal cycles but also can be influenced by psychological factors and might reflects differences in subjective awareness of the illness to motion and other nauseogenic stimuli.(Golding et al., 2005).

Anatomical evidence supports the view that vestibular stress such as motion sickness may modulate hypothalamic-pituitary-adrenal (HPA) axis through the vestibulo-paraventricular pathways (Markia et al., 2008). The present study also found that female animals had significantly higher defecation responses after rotation than males did. It seems that females tend to show heightened stress responses than males after rotation (Curtis et al., 2006; Goel and Bale, 2009). However, we revealed that rotation only significantly affected blood ACTH and corticosterone levels in male young adults, and significant gender difference on blood hormone responses after rotation was observed across ages. Although, we did not observe hormone and defecation responses as a function of time during rotation, there is sufficient evidence supporting that temporal changes of blood stress hormones did not synchronize those of motion-induced nausea, and that the activation of HPA axis might be a transient response to provocative motion rather than a causing factor of motion-induced autonomic responses (Otto et al., 2006). In addition, it is also noteworthy that female animals always exhibited greater behavioral responses to stress, such as increased immobile time in the forced swim and tail suspension tests, compared to males (Bale and Vale, 2003; Altemus, 2006). Similarly, the present study also found that there were great sex differences in spontaneous locomotion activity in middle-aged animals. However, motion sickness can induce neurosensory, sensory-motor and perceptual disturbances which might also impare motor performance immediately after the cessation of motion stimulation (Reschke et al., 1998). Recent studies have confirmed that spatial orientation preference had a significant relationship with susceptibility to simulated motion sickness symptoms (Chen et al., 2015, 2016). These evidence suggest that sex differences in motion sickness induced locomotion disorder might also be associated with the diversity in spatial cognition and spatial orientation (Bettis and Jacobs, 2009; Locklear and Kritzer, 2014). Furthermore, balance performance in females was not different from males during training period in the present study. Male and female animals exhibited almost equivalent perturbations in motor coordination after rotation during adolescent and young adulthood. Although there are great differences in the spatial magnitude of postural sway and the control of posture in humans (Koslucher et al., 2016a,b), our results suggest that quadrupeds (e.g., rodents) seem to be more stable than bipeds (e.g., humans) in both anteroposterior and mediolateral body axes and postural sway might not be a common factor determining motion sickness susceptibility across species (Takahashi et al., 1991).

The present study also found that rotation-induced gastrointestinal responses (conditioned gaping and defecation responses) declined during aging in both males and females. These results are consistent with previous studies which showed that rotation-induced pica decreased with age in rodents (McCaffrey and Graham, 1980), and middle-aged and aged subjects were more resistant to motion sickness than adolescents and young adults (Cooper et al., 1997; Gahlinger, 2000). Since animals had never received motion challenges before experiment, it clearly suggested that the age-related differences are mainly attributed to physiological aging process but not habituation that was commonly seen in humans. In the meantime, the age-related changes in blood levels of ACTH, corticosterone, norepinephrine and epinephrine are also consistent with previous findings (Hauger et al., 1994; Ivanisevic-Milovanovic et al., 1997). However, these changes might not contribute to age differences in motion sickness due to a lack of age × rotation interaction effects. In addition, many studies have found an age-related degeneration of the peripheral vestibular receptor hair cells, the Scarpa’s ganglion neurons and vestibular nucleus neurons in humans and some animal species (Brosel et al., 2016; Smith, 2016). Some studies also found structural changes in cytoplasm such as nuclear membrane invaginations, disorganized endoplasmic reticulum and lipofuscin-like dense bodies in vestibular nucleus neurons of rodents (Takeuchi et al., 1997; Fernandez et al., 2007). However, our study found that rotation-induced Fos expression was significantly increased in the SpVN, the NTS and the PBN as well as in the CeA and the MeA in an age-dependant manner, indicating that neuronal activity in these vestibular and autonomic nuclei might not be influenced by the pathological changes during aging. Our observations also coincide with previous findings regarding age-related differences in central Fos expression. For example, adult mice treated with lipopolysaccharide (LPS) display significantly greater Fos immunoreactivity compared to their saline controls while adolescent mice show no difference in Fos expression between LPS- and saline-treated mice (Girard-Joyal et al., 2015). Pubertal rats also showed reduced Fos expression in several brain regions compared to adult animals following a 2 h restraint stress (Kellogg et al., 1998). Meanwhile, it is widely accepted that the caudal aspect of vestibular nuclei including the MVN and the SpVN participates in generating motion-induced autonomic responses in rodents (Yates et al., 2014). Our previous study showed that direct pathways from the MVN and the SpVN to the NTS and the PBN were specifically activated by rotation stimulation in adult rats (Cai et al., 2007). Recent studies have demonstrated that the PBN was necessary for the anorexigenic effects of LiCl and for CTA acquisition in mice and the NTS-PBN-CeA connections mediated cisplatin-induced pica, anorexia and loss of body weight in rats (Alhadeff et al., 2015; Carter et al., 2015; Roman et al., 2016). Meanwhile, cisplatin injection or hypergravity stimulation also increased Fos expression in rat CeA and MeA and in the ferrets receiving Exendin-4 injection (Nakagawa et al., 2003; Horn et al., 2007; Lu et al., 2016). Functional magnetic resonance imaging studies found that the amygdala was activated in humans experiencing motion sickness-induced nausea or mal de debarquement syndrome (Cha et al., 2012; Napadow et al., 2013). Moreover, our study also found that the middle-aged animals had much greater decline in the rotation-induced conditioned gaping reactions than the LiCl-induced ones. Middle-aged males with greater reduction in locomotion responses after rotation also had lower numbers of Fos-LI neurons in the NTS, the PBN, as well as in the LC and the PVN than female animals. These evidences strongly support the viewpoint that increased Fos expression in these vestibular and autonomic nuclei might correlate with the decreased rotation-related behavioral responses during aging in the rats. However, whether these activated neurons are involved in regulation of intrinsic “homeostasis” after motion stimulation is still unclear and needs further investigation (Martinez et al., 2002).

Our previous study found that rotation stimulation significantly reduced both horizontal and vertical spontaneous motor activity in rats, but the change of horizontal movement seemed to be more pronounced than the vertical movement (Wang et al., 2012). In contrast, rats with chemically induced vestibular dysfunction exhibited more open-field ambulation but fewer rearing responses compared with controls (Ossenkopp et al., 1990, 1992). The current spontaneous locomotion observation also coincides with previous results showing hypoactivity in rodent motion sickness model (Ossenkopp et al., 1994; Santucci et al., 2009). In addition, the present study revealed an age-related alleviation in rotation-induced motor disorders (i.e., hypoactivity and balance disturbance) in the rats. The results of balance beam test are inconsistent with a previous observation showing that the impact of rotation stimulation (without inducing overt signs of motion sickness) on balance beam performance is significantly greater in 9-month-old and 13-month-old mice than in 1-month-old mice (Tung et al., 2014). The discrepancy can be explained mainly by several factors such as species, rotation pattern and training procedure. As we observed similar age-related alterations in males and females, it is reasonable to conclude that the impact of age on vestibular stimulation-induced motor disorders might possibly depend on whether or not the motion sickness has been triggered. Furthermore, our results also suggested that the SpVN might play a role in age dependent motor disorders-induced by motion sickness. This notion can be supported by the fact that medial vestibulospinal tracts in part originates from the SpVN in rodents (Liang et al., 2015). Immunohistochemical studies confirmed the existence of an inhibitory influence of SpVN to the spinal cord (Blessing et al., 1987; Valla et al., 2003). Further investigations should be performed to identify the precise neuron population in the SpVN that is involved in age-related regulation of autonomic and/or locomotor responses during motion sickness.

In summary, we found an age-related alleviation in motion sickness-related gastrointestinal responses (nausea and defecation) and motor disorders (hypoactivity and balance disturbance) especially in middle-aged rats. Females only had higher defecation responses during adolescent and young-adult period than males. Although there were significant age-related alterations in several blood hormones (ACTH, corticosterone, norepinephrine, and epinephrine), such effects are irrelevant to rotation in both males and females. The present study also found remarkable age differences in Fos expression pattern in nausea-related brain structure including the SpVN, the NTS, the PBN, and the amygdala, with a significant increase in Fos levels in the middle-aged animals compared with the adolescents and the young-adults. Females showed higher Fos levels in the NTS, the PBN, the LC, and the PVN during young-adult and/or middle-aged period than males. These findings provide the evidence that the sex and age differences in provocative motion-induced gastrointestinal responses and motor disorders may not correlate with stress hormone responses and habituation. The age-related decline in motion sickness susceptibility might be mainly attributed to the activity changes in neurons of the vestibular and autonomic areas including the SpVN, the NTS, the PBN, and the amygdala.

## Author Contributions

WZ, JW, LP, and RQ performed behavioral experiments. WZ, JW, and LP were responsible for the stress hormone measurements. LP, WZ, and JW completed immunohistochemistry experiment. JL and PL were responsible for animal breeding. WZ, JW, and YC were responsible for the design of the work. LP, PL, and WZ were responsible for data analysis. YC, WZ, and JW were responsible for writing the manuscript. All authors read and approved the final manuscript.

## Conflict of Interest Statement

The authors declare that the research was conducted in the absence of any commercial or financial relationships that could be construed as a potential conflict of interest.
